# Machine learning-based prediction of proximal junctional pathology after adult spinal deformity surgery: a systematic review and diagnostic test accuracy meta-analysis

**DOI:** 10.1007/s00701-026-06876-6

**Published:** 2026-05-07

**Authors:** Shaan Patel, Shiva A. Nischal, Yi Hein Chai, Kush M. Kale, Kevin Hines, Joshua Heller, Jack Jallo, James S. Harrop, Srinivas K. Prasad

**Affiliations:** 1 Department of Neurological Surgery, Thomas Jefferson University Hospital, Philadelphia, PA USA; 2 Department of Physiology, Anatomy & Genetics, Medical Sciences Division, University of Oxford, Oxford, UK; 3 University College London Medical School, University College London, London, UK

**Keywords:** Proximal junctional kyphosis, Proximal junctional failure, Adult spinal deformity, Machine learning, Artificial intelligence, Predictive modelling

## Abstract

**Background:**

Proximal junctional kyphosis (PJK) and failure (PJF) remain challenging and incompletely predictable adverse event following adult spinal deformity (ASD) surgery. Machine learning (ML) models have been proposed to improve risk stratification, but performance varies and clinical applicability remains uncertain. A systematic review and diagnostic test accuracy meta-analysis was performed to synthesize the predictive performance of ML-based models for proximal junctional pathology after ASD surgery.

**Methods:**

PubMed, Embase, and CENTRAL were searched from inception to February 2026. Studies developing or validating ML models predicting PJK or PJF following ASD surgery were included. Diagnostic performance was synthesized using random-effects models, pooling sensitivity, specificity, diagnostic odds ratio (dOR), and summary receiver operating characteristic (SROC) curves. A prespecified secondary analysis pooled one highest-performing model per study to reduce within-study dependence. Risk of bias was assessed using PROBAST and certainty of evidence using GRADE.

**Results:**

Sixteen studies comprising 3625 patients and 30 ML models met inclusion criteria. Twenty-two models provided extractable test-set data for diagnostic synthesis. Pooled sensitivity was 0.51 (95% CI: 0.41–0.61) and specificity 0.84 (95% CI: 0.78–0.89), with a pooled AUC of 0.67 (95% CI: 0.62–0.72). In the highest-performing model analysis, pooled sensitivity improved to 0.63 (95% CI: 0.55–0.71) and AUC to 0.77 95% CI: 0.71– 0.83). Specificity remained high (0.86). External validation was rare, and calibration was inconsistently reported. Frequently prioritized predictors included sagittal alignment parameters, construct characteristics, bone quality metrics, and age.

**Conclusions:**

ML-based models demonstrate biologically coherent but clinically moderate discrimination for predicting proximal junctional pathology following ASD surgery. Current performance supports risk stratification rather than categorical decision-making. Progress toward clinically actionable prediction will require standardized endpoint definitions, robust external validation, and calibration-centered reporting.

**Supplementary Information:**

The online version contains supplementary material available at 10.1007/s00701-026-06876-6.

## Introduction

Adult spinal deformity (ASD) surgery seeks to improve quality of life through restoration of global alignment, decrease pain, and improve functional capacity [1, 8, 18, 30]. Although contemporary techniques provide substantial radiographic correction, long-segment constructs often introduce an abrupt transition between rigid spinal constructs and the mobile adjacent spine. This mechanical discontinuity may predispose the proximal junction to progressive kyphotic deformation and result in structural breakdown [30, 41, 43, 44].

Proximal junctional kyphosis (PJK) and proximal junctional failure (PJF) represent related but clinically distinct manifestations along this spectrum [11, 21, 30]. PJK is typically defined by radiographic parameters such as an increase in proximal junctional angle (PJA) relative to baseline, whereas PJF reflects structural compromise at or adjacent to the upper instrumented vertebra (UIV), frequently accompanied by pain, neurologic sequelae, and the need for revision surgery [9, 21, 36]. Despite extensive analysis, definitions remain heterogeneous, and reported incidence of PJK varies widely. Unfortunately, the ability to predict which patients will progress from radiographic angular change to clinically significant failure remains limited.

Traditional regression-based models have identified associations spanning demographic factors, sagittal alignment, construct characteristics, and bone quality [12, 19]. However, these approaches rely on linear assumptions and population-average effects that may incompletely capture the multi-level interactions underlying junctional failure [23, 26]. Mechanistically, PJK and PJF likely emerge from nonlinear interplay between baseline alignment demands, magnitude of surgical correction, construct stiffness gradients, vertebral strength, and soft tissue reserve. Such complexity has prompted growing interest in artificial intelligence (AI) and machine learning (ML) approaches capable of modelling high-dimensional feature interactions without prespecified assumptions.

Over the past decade, ML-based predictive models have been proposed to estimate risk of PJK and PJF after ASD surgery [23, 26, 37]. These models vary considerably in architecture, input features, endpoint definitions, and validation strategy. Reported discrimination ranges from modest to apparently excellent. External validation remains rare, calibration is inconsistently reported, and thresholds for clinical decision-making are seldom standardized. Furthermore, some models incorporate postoperative or early follow-up variables, blurring the distinction between preoperative risk stratification and early trajectory prediction.

As a result, uncertainty persists regarding the true diagnostic performance and clinical readiness of ML-based prediction in this domain. To date, no study has systematically synthesized ML model accuracy for PJK and PJF using a diagnostic test accuracy framework.

Accordingly, we performed a systematic review and diagnostic test accuracy meta-analysis of ML-based prediction models for PJK and PJF following ASD surgery. Our objectives were three-fold: (i) to quantify pooled sensitivity, specificity, and global discrimination; (ii) to characterize feature domains, validation strategies, and interpretability reporting; (iii) to evaluate methodological quality and translational readiness. By situating ML performance within a diagnostic accuracy framework, we aim to clarify whether current models offer meaningful clinical risk stratification or primarily represent exploratory computational efforts requiring further validation.

## Materials and methods

This systematic review and diagnostic test accuracy meta-analysis was conducted according to PRISMA-DTA [27] and AMSTAR-2 [42] guidance. The protocol was registered prospectively on PROSPERO (CRD420261308618).

### Search strategy

We searched PubMed, Embase, and CENTRAL from inception to 14 February 2026, using controlled vocabulary and free-text terms related to AI, ML, ASD, PJK, and PJF (Supplementary Table 1). Searches were limited to human, English-language studies. Reference lists of eligible studies and relevant reviews were screened to identify additional records.

### Eligibility criteria

Studies were included if they: (i) enrolled patients undergoing ASD surgery; (ii) developed or validated a ML model predicting PJK or PJF; (iii) reported extractable model performance data, including area under the curve (AUC), or sufficient information to derive sensitivity and specificity (such as event and non-event counts in the test set or confusion matrix elements).

We excluded studies that did not use ML for PJK/PJF prediction, did not report extractable performance data, were case series with < 10 patients, conference abstracts, editorials/commentaries, or were non-English publications.

### Study selection

Two reviewers independently screened titles/abstracts and full-texts using Rayyan [31]. Disagreements were resolved by consensus with third reviewer adjudication. Inter-rater agreement was quantified using Cohen’s kappa (κ = 0.91). References of included studies were manually searched to identify additional eligible studies.

### Data extraction and definitions

For this analysis, PJK was defined radiographically as a study-specific increase in PJA relative to baseline, whereas PJF was defined as structural failure at or adjacent to the UIV, including vertebral fracture, posterior osseoligamentous disruption, or implant failure, and frequently accompanied by clinical deterioration or revision surgery.

Using a standardized template, two reviewers independently extracted: study and cohort characteristics (country, design, sample size, follow-up, inclusion criteria, ASD definitions); outcome definitions (recorded exactly as specified by each study); model characteristics (algorithm type, input modality, feature engineering, cross-validation strategy, train/test/validation split, feature selection or importance approach, and reporting of calibration and interpretability methods); feature domains for synthesis (predictors mapped a priori into patient factors, operative/construct factors, bone quality proxies, radiographic/alignment parameters, and sarcopenia/muscle metrics); diagnostic accuracy data (test-set and external validation-set event and non-event totals and confusion matrix data).

To distinguish model development from true validation performance, analyses preferentially used held-out test and external validation when available. Training and cross-validation metrics were extracted for descriptive reporting only.

### Statistical analysis

Primary analyses synthesized model discrimination as a diagnostic test accuracy problem. Pooled sensitivity and specificity were estimated using random-effects (Hartung-Knapp) models. Overall performance was summarized using summary receiver operating characteristic (SROC) methods with corresponding pooled AUCs, where supported by the data structure. From confusion matrix data, pooled positive likelihood ratios (PLRs), negative likelihood ratios (NLRs), and diagnostic odds ratios (dORs) were calculated with 95% confidence intervals (CIs). Statistical significance was set at α = 0.05.

Only models with extractable test-set or external validation confusion matrix data were pooled, and models reporting only training or cross-validation performance were excluded. As several studies report multiple models derived from the same cohort, a prespecified secondary analysis pooled one model per study (defined as the highest performing model by AUC) to reduce within-study dependence and provide a clinically interpretable estimate of “best available” performance.

All analyses were conducted in R version 4.3.2 [34] using appropriate meta-analysis and diagnostic accuracy packages.

### Risk of bias and certainty of evidence

Risk of bias and applicability were assessed using PROBAST [49] across four domains (participants, predictors, outcome, analysis), with disagreements resolved by consensus. Certainty of evidence for pooled diagnostic outcomes was evaluated using GRADE [39] adapted for prediction model performance.

### Small-study effects and clinical utility

Small-study effects were evaluated using Deeks’ funnel plot asymmetry test where feasible. Clinical utility was explored using Fagan nomogram, translating pooled likelihood ratios into post-test probabilities based on observed event prevalence.

## Results

### Study selection and included evidence

**Fig. 1:**
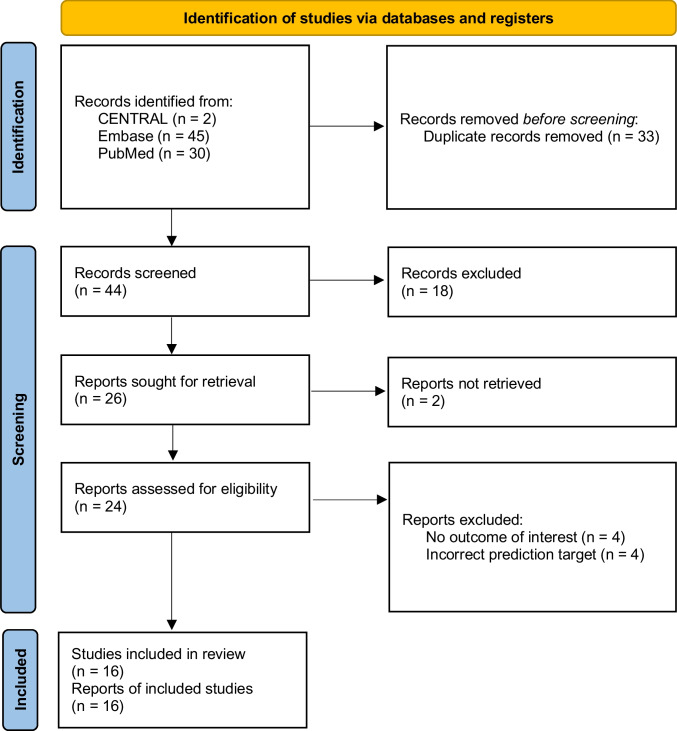
PRISMA flow diagram of study selection. Preferred Reporting Items for Systematic Reviews and Meta-Analyses (PRISMA) flow diagram outlining the study selection process. The number of records identified, screened, assessed for eligibility, and included in the final analysis are detailed at each stage

The search identified 77 records. After duplicate removal and screening, 16 retrospective cohort studies were included [12, 13, 15, 17, 22, 24, 25, 28, 32, 33, 35, 38, 45, 46, 48, 50], comprising 3625 patients and 30 ML models predicting proximal junctional pathology after ASD surgery (Fig. 1).

### Study and cohort characteristics

Study-level characteristic outcome definitions and inclusion criteria are detailed in Table 1, baseline cohort features by outcome group in Table 2, and surgical construct characteristics in Table 3.

**Table 1:** Study characteristics, outcome definitions, and inclusion criteria of machine learning studies predicting proximal junctional pathology

Author (year)	Study design	Country	Follow-up (months)	Population size (*n*)			Event rate (%)	Definitions & criteria			PJA measurement (Sagittal Cobb angle)	Inclusion criteria	Primary outcome predicted
				PJK/PJF	Non-PJK/PJF	Total		ASD	PJK	PJF			
Scheer (2016)	RC	USA	24.0	139	371	510	27.25	Cobb angle ≥ 20° SVA ≥ 5 cm PT ≥ 25° TK ≥ 60°	≥ Δ20° PJA from baseline ≥ 1 SRS-Schwab modifier deterioration (6-week postoperatively)	Revision required for PJK	Between UIV (inferior endplate) and UIV + 2 (superior endplate)	≥ 18 years ≥ 1 ASD criterion met ≥ 4 vertebral levels fused ≥ 2-year follow-up	PJK/PJF incidence
Yagi (2018)	RC	Japan	24.0	22	90	112	19.64	Cobb angle ≥ 20° SVA (C7) ≥ 5 cm PT ≥ 25°	NR	≥ Δ20° PJA from baseline ≥ 1 SRS-Schwab modifier deterioration (immediate postoperatively) Revision surgery	NR	> 50 years ≥ 1 ASD criterion met > 5 vertebral levels fused ≥ 2-year follow-up	PJF incidence
Johnson (2023)	RC	USA	≥ 24.0	89	102	191	46.6	Affecting QoL	> 10° PJA > Δ10° PJA from baseline	NR	Between UIV and UIV + 2	≥ 5 vertebral levels fused ≥ 2-year follow-up Preoperative X-ray	PJK incidence
Lee (2023)	RC	South Korea	≥ 12.0	49	152	201	24.4	SVA ≥ 5 cm PT ≥ 25° PI–LL mismatch > 10° TK ≥ 60°	≥ 20° PJA (last follow-up) ≥ Δ10° PJA from baseline	NR	Between UIV (inferior endplate) and UIV + 2 (superior endplate)	≥ 18 years ≥ 1 ASD criterion met ≥ 1-year follow-up	PJK incidence
Ryu (2023)	RC	South Korea	> 24.0	58	152	210	39.5	Cobb angle ≥ 20° SVA ≥ 5 cm PT ≥ 25° TK ≥ 60°	> 10° PJA	Symptomatic PJK ≥ 15° PJA Structural compromise Revision surgery	Between UIV and UIV + 2	≥ 4 vertebral levels fused ≥ 2-year follow-up	Unplanned reoperation due to PJK/PJF
Tretiakov (2023)	RC	USA	12.0	391	388	779	50.20 (PJK); 10.50 (PJF)	Cobb angle ≥ 20° SVA ≥ 5 cm PT ≥ 25° TK ≥ 60°	≥ 10° PJA	≥ 15° PJA Structural compromise Revision surgery	Between UIV (inferior endplate) and UIV + 2 (superior endplate)	≥ 1 ASD criterion met Preoperative X-ray & HR-QoL 6-week follow-up X-ray & HR-QoL 2-year follow-up X-ray & HR-QoL	PJK/PJF incidence
Hiyama (2025)	RC	Japan	25.5 ± 14.9	21	71	92	22.8	Cobb angle ≥ 20° SVA ≥ 5 cm PT ≥ 25° TK ≥ 60°	NR	Structural compromise Revision surgery	NR	≥ 6 vertebral levels fused (LIV at sacrum) ≥ 1-year follow-up	PJF incidence
Meng (2025)	RC	China	≥ 24.0	24	118	142	16.9	NR	≥ 10° PJA ≥ Δ10° PJA from baseline	Fracture, implant failure, or PJK requiring cranial extension of the fusion cranially	NR	> 50 years ≥ 1 ASD criterion met ≥ 2-year follow-up Preoperative CT & MRI	PJK incidence
Sundrani (2025)	RC	USA	≥ 24.0	147	84	231	63.6	SVA ≥ 5 cm CVA ≥ 3 cm PT ≥ 25° TK ≥ 60°	≥ Δ10° PJA from baseline	> Δ10° PJA from baseline Structural compromise	Between UIV and UIV + 2	≥ 18 years ≥ 2 ASD criterion met ≥ 5 vertebral levels fused ≥ 2-year follow-up Elective surgery	MC (PJK/PJF composite)
Wang (2025)	RC	China	> 24.0	78	135	213	36.6	NR	> 20° PJA > Δ10° PJA from baseline	NR	Between UIV (inferior endplate) and UIV + 2 (superior endplate)	≥ 2-year follow-up	PJK incidence
Li (2025)	RC	China	31.0 ± 5.8 (non-MC); 30.4 ± 5.6 (MC)	101	112	213	47.42	Cobb angle ≥ 20° SVA ≥ 5 cm PT ≥ 25° TK ≥ 60°	≥ 10° PJA ≥ Δ10° PJA from baseline	Fracture, implant failure, or PJK requiring cranial extension of the fusion cranially	NR	≥ 50 years ≥ 1 ASD criterion met	MC (PJK/PJF composite)
Pizones (2024)	RC	Spain	57.6 ± 21.6	PJK: 46 PJF: 21	324	391	17.3	Cobb angle ≥ 20° SVA ≥ 5 cm PT ≥ 25° TK ≥ 60°	> Δ25° PJA (last follow-up) from immediate postoperative	Fracture, implant failure, pull-out or dislodgement, proximal spondylolisthesis	NR	> 18 years ≥ 1 ASD criterion met	PJK/PJF incidence, MC (PJK/PJF composite)
Li (2023)	RC	China	≥ 24.0	53	60	113	46.9	NR	≥ 10° PJA ≥ Δ10° PJA from baseline	NR	Between UIV (inferior endplate) and UIV + 2 (superior endplate)	≥ 50 years ≥ 2-year follow-up ≥ 3 timepoints imaged Posterior surgery	PJK incidence
Kim (2020)	RC	South Korea	21.8 ± 16.6	32	38	70	45.71	NR	≥ 10° PJA ≥ Δ10° PJA from baseline	NR	Between UIV (inferior endplate) and UIV + 2 (superior endplate)	≥ 5 vertebral levels fused ≥ 2-year follow-up	PJK recurrence
Peng (2020)	RC	China	≥ 24.0	3	9	12	25	NR	≥ 10° PJA ≥ Δ10° PJA from baseline	NR	Between UIV (inferior endplate) and UIV + 2 (superior endplate)	≥ 2-year follow-up	PJK incidence
Hills (2022)	RC	USA	26.5 (24.0–48.7)	47	98	145	32.4	Cobb angle ≥ 20° SVA ≥ 5 cm PT ≥ 25° TK ≥ 60°	≥ 10° PJA ≥ Δ10° PJA from baseline	NR	Between UIV (inferior endplate) and UIV + 2 (superior endplate)	≥ 18 years ≥ 1 ASD criterion met ≥ 5 vertebral levels fused ≥ 2-year follow-up	PJK incidence, severity score (ISSG)

Summary of included retrospective cohort (RC) studies evaluating machine learning (ML) models for prediction of proximal junctional kyphosis (PJK), proximal junctional failure (PJF), or composite mechanical complications (MC) following adult spinal deformity (ASD) surgery. For each study, country of origin, follow-up duration, cohort size (PJK/PJF, non-PJK/PJF, and total), event rate, radiographic definitions of ASD and proximal junctional pathology, proximal junctional angle (PJA) measurement method, inclusion criteria, and primary outcome predicted are reported. Where applicable, PJA was calculated as the sagittal Cobb angle between the inferior endplate of the upper instrumented vertebra (UIV) and the superior endplate of UIV+2. “NR” denotes not reported

*ASD* Adult spinal deformity, *BMI* Body mass index, *Cobb* Cobb angle, *CT* Computed tomography, *CVA* Coronal vertical axis, *HR-QoL* Health-related quality of life, *ISSG* International Spine Study Group, *LIV* Lower instrumented vertebra, *MC* Mechanical complication, *MRI* Magnetic resonance imaging, *PJA* Proximal junctional angle, *PJF* Proximal junctional failure, *PJK* Proximal junctional kyphosis, *PI* Pelvic incidence, *PI–LL* Pelvic incidence–lumbar lordosis mismatch, *PT* Pelvic tilt, *QoL* Quality of life, *RC* Retrospective cohort, *SRS-Schwab* Scoliosis Research Society–Schwab classification, *SVA* Sagittal vertical axis, *TK* Thoracic kyphosis, *UIV* Upper instrumented vertebra

**Table 2:** Baseline demographic and radiographic characteristics stratified by outcome group

Author (year)	Age (years) (mean ± SD or range (mean))		Female (%)		BMI (kg/m^2^) (mean ± SD or range (mean))		Baseline CT-HU (mean ± SD or range (mean))		Baseline SVA (mean ± SD or range (mean))		Baseline PT (mean ± SD or range (mean))		Baseline PI-LL (mean ± SD or range (mean))		Baseline PI (mean ± SD or range (mean))		Baseline SRS-Schwab coronal curve type (%)				Baseline SRS-Schwab global balance modifier (%)		
	PJK/PJF	Non- PJK/PJF	PJK/PJF	Non- PJK/PJF	PJK/PJF	Non- PJK/PJF	PJK/PJF	Non- PJK/PJF	PJK/PJF	Non- PJK/PJF	PJK/PJF	Non- PJK/PJF	PJK/PJF	Non- PJK/PJF	PJK/PJF	Non- PJK/PJF	Type N	Type T	Type L	Type D	PT	SVA	PI-LL
Scheer (2016)	63.30 ± 10.90	54.90 ± 14.20	77.7		27.3 ± 5.9		NR		110.2 ± 88.4	78.8 ± 88.5	28.5 ± 9.6	25.6 ± 13.1	24.5 ± 23.3	18.8 ± 25.8	NR		42.5	4.0	32.3	21.2	0 (29.2 %) + (35.1 %) ++ (35.7 %)	0 (33.8 %) + (21.8 %) ++ (44.4 %)	0 (33.5 %) + (15.3 %) ++ (51.2 %)
Yagi (2018)	63.90 ± 9.40		96.0		21.9 ± 3.9		NR		8.2 ± 6.1		30.9 ± 11.6		35.9 ± 22.6		NR		28.0	8.0	46.0	18.0	NR		
Johnson (2023)	65.90 ± 16.30	60.70 ± 19.80	82.0	71.6	29.3 ± 6.7	28.3 ± 7.2	NR		81.4 ± 72.7	61.2 ± 64.8	27.9 ± 12.2	23.4 ± 11.4	23.6 ± 22.9	17.6 ± 22.4	55.1 ± 16.6	52.1 ± 16.6	NR				NR		
Lee (2023)	67.00 ± 7.61	67.21 ± 9.53	78.6		23.6 ± 4.8	25.1 ± 3.6	NR		83.6 ± 66.5	78.5 ± 73.0	29.5 ± 16.4	29.9 ± 12.5	NR		48.8 ± 13.2	51.3 ± 11.3	51.2	1.5	9.5	31.8	0 (15.1 %) + (30.3 %) ++ (51.1 %)	0 (24.3 %) + (28.3 %) ++ (38.8 %)	0 (11.2 %) + (30.3 %) ++ (68.4 %)
Ryu (2023)	66.90 ± 6.60	68.90 ± 8.70	85.2		25.0 ± 3.7		NR		15.5 ± 20.1)	13.4 ± 14.4	32.6 ± 11.9	32.9 ± 12.3	NR		NR		NR				NR		
Tretiakov (2023)	65.41 ± 8.47	58.83 ± 15.48	78.0		28.2 ± 4.7	28.0 ± 6.1	NR		21.0 ± 46.3	32.0 ± 48.9	20.8 ± 11.4	19.9 ± 9.7	2.3 ± 14.6	4.8 ± 13.6	54.8 ± 13.2	57.1 ± 13.6	NR				NR		
Hiyama (2025)	71.10 ± 8.40		90.0		22.6 ± 3.8		NR		160.0 ± 71.0		33.7 ± 9.5		43.0 ± 22.3		49.8 ± 9.1		NR				NR		
Meng (2025)	68.70 ± 6.70	67.30 ± 6.50	66.9		25.3 ± 6.3	25.8 ± 3.8	NR		38.4 ± 25.6	36.3 ± 21.0	23.1 ± 10.9	22.3 ± 9.8	NR		45.8 ± 10.8	50.7 ± 13.2	NR				NR		
Sundrani (2025)	66.00 ± 14.70	59.40 ± 20.70	76.2		NR		NR		50.7 ± 58.9		24.6 ± 10.7		NR		NR		NR				NR		
Wang (2025)	72.00 (67.00–76.00)		72.3		25.5 (22.3–27.9)		NR		101.8 (72.8–142.1)	114.0 (72.8–154.7)	22.9 (18.1–31.1)	29.5 (23.0–34.7)	26.1 (20.3–36.7)	32.8 (20.8–39.1)	47.9 ± 10.0	49.4 ± 8.8	NR				NR		
Li (2025)	61.80	60.10	87.1	83.9	24.7 ± 3.3	24.8 ± 3.1	NR		5.9 ± 5.4	3.4 ± 4.5	28.7 ± 12.9	23.8 ± 11.3	NR		47.9 ± 15.6	45.6 ± 14.5	NR				NR		
Pizones (2024)	65.00 ± 10.20		82.1		26.1 ± 4.5		NR		NR		NR		NR		57.9 ± 13.7		NR				NR		
Li (2023)	61.72 ± 9.57	59.98 ± 9.23	83.0		25.9 ± 4.7	25.1 ± 3.5	NR		43.0 ± 5.5	50.0 ± 5.1	26.6 ± 12.0	23.2 ± 12.7	27.4 (12.8–51.7)	35.5 (15.4–68.7)	48.9 ± 15.7	48.9 ± 11.0	NR				NR		
Kim (2020)	66.50 ± 7.80	65.80 ± 11.40	80.0		NR		NR		63.1 ± 65.2		26.4 ± 10.5		5.7 ± 17.3		NR		NR				NR		
Peng (2020)	17.33	15.56	66.7		NR		50.0–150.0		NR		NR		NR		NR		NR				NR		
Hills (2022)	66.20 (59.80–71.00)	28.80 (24.90–33.00)	81.4		28.8 (24.9–33.0)		139.3 (120.6–180.1)		81.0 (34.0–121.0)		25.8 (19.7–31.9)		20.3 (9.2–35.0)		50.9 (43.5–59.7)		NR				0 (28.7%) + (40.6%) ++ (30.8%)	0 (28.6%) + (30.1%) ++ (41.4%)	0 (23.8%) + (21.7%) ++ (54.5%)

Baseline patient demographics, bone quality metrics, and preoperative radiographic parameters across included studies, stratified by proximal junctional kyphosis (PJK), proximal junctional failure (PJF), or composite mechanical complication status where reported. Continuous variables are presented as mean ± standard deviation (SD) or median (range/interquartile range) as originally reported. Radiographic alignment parameters include sagittal vertical axis (SVA), pelvic tilt (PT), pelvic incidence–lumbar lordosis mismatch (PI–LL), pelvic incidence (PI), and SRS-Schwab coronal curve type and global balance modifiers. Where applicable, SRS-Schwab modifiers are reported as 0 (no deformity), + (moderate deformity), or ++ (severe deformity). “NR” denotes not reported

*ASD* adult spinal deformity; *BMI* body mass index; *CT-HU* computed tomography Hounsfield units; *LL* lumbar lordosis; *MC* mechanical complication; *PI* pelvic incidence; *PI–LL* pelvic incidence–lumbar lordosis mismatch; *PJF* proximal junctional failure; *PJK* proximal junctional kyphosis; *SD* standard deviation; *SRS-Schwab* Scoliosis Research Society–Schwab classification; *SVA* sagittal vertical axis; *TK* thoracic kyphosis

**Table 3:** Surgical characteristics and construct distribution across included studies

Author (year)	Surgery (n)		Fused levels (n) (mean ± SD or mean (range))		UIV distribution (%)		LIV distribution (%)		3-colum osteotomy (%)		Type of implant (%)	Surgical approach (%)	
	Primary	Revision	PJK/PJF	Non-PJK/PJF	PJK/PJF	Non-PJK/PJF	PJK/PJF	Non-PJK/PJF	PJK/PJF	Non-PJK/PJF		PJK/PJF	Non-PJK/PJF
Scheer (2016)	200	310	11.8 ± 3.7		C–S (1.4) T1–T5 (48.6) T6–T9 (12.0) T10–L3 (38.0)		T11–L2: (6.1) L3–L5 (15.7) Sacroiliac (78.2)		59.7	55.5	Hooks (17.6) Screw (82.4)	NR	
Yagi (2018)	101	12	9.9 ± 2.3		NR		Pelvis (55.0) ≥ L5 (45.0)		NR		NR	NR	
Johnson (2023)	135	56	10.6 ± 3.0		T1 (0.5) T2 (4.2) T3 (7.9) T4 (14.1) T5 (7.9) T6 (2.6) T7 (3.1) T8 (9.4) T9 (9.9) T10 (23.0) T11 (7.9) T12 (5.8) L1 (0.5) L2 (3.1)		NR		NR		NR	NR	
Lee (2023)	145	56	7.1 ± 3.0		NR		Ileal (35.3) S2–alar (2.5) S2–alar–ileal (5.5)		6.1	2.0	NR	NR	
Ryu (2023)	152	58	7.3 ± 2.2	7.8 ± 2.1	NR		NR		NR		NR	NR	
Tretiakov (2023)	NR		12.6 ± 3.7	9.5 ± 4.8	NR		NR		23.5	16.5	NR	Combined (24.5) Posterior (75.5) Anterior (0.0)	Combined (35.2) Posterior (63.9) Anterior (0.9)
Hiyama (2025)	NR		9.2 ± 2.3		T1–5 (15.2) T6–8 (9.8) T9–12 (72.8) L1–2 (2.2)		Sacral (100.0)		NR		NR	Combined (100.0)	
Meng (2025)	NR		5.0 ± 1.9	5.2 ± 2.2	NR		NR		NR		NR	Posterior (100.0)	
Sundrani (2025)	NR		NR		NR		NR		NR		NR	NR	
Wang (2025)	NR		7.0 (6.0–9.0)		T1–5 (5.2)		NR		31.5		NR	NR	
Li (2025)	NR		9.7 ± 2.6	8.8 ± 2.6	> T8 (25.7) T8–10 (56.4) < T10 (17.8)	> T8 (25.0) T8–10 (31.3) < T10 (43.8)	NR		NR		NR	NR	
Pizones (2024)	210	181	11.2 ± 3.4		T1–T6 (24.7%), T8–L1 (75.3%)		NR		NR		NR	Combined (11.0) Posterior (89.0)	
Li (2023)	NR		NR	> T10 (32.1) T11–L1 (30.2) < L2 (37.7)	> T10 (31.0) T11–L1 (29.3) < L2 (39.7)	> L5 (37.7) S1 (62.3)	> L5 (41.8) S1 (50.0)		NR		NR	NR	
Kim (2020)	0	70	NR	T1–5 (25.7) T6–12 (74.3)		Sacroiliac (94.3)			NR		NR	NR	
Peng (2020)	9	3	NR	NR		NR			NR		Screws (100.0)	NR	
Hills (2022)	20	27	9.0 (8.0–11.0)	T7–L2 (80.7)		NR			NR		Hooks (5.5)	NR	

Summary of operative characteristics for included cohorts, including number of primary and revision surgeries, fusion length, upper instrumented vertebra (UIV) and lower instrumented vertebra (LIV) distribution, three-column osteotomy (3-CO) rates, implant type, and surgical approach. Where reported, values are stratified by proximal junctional kyphosis (PJK), proximal junctional failure (PJF), or composite mechanical complication status. Fusion length is presented as mean ± standard deviation (SD) or median (range) as originally reported. UIV and LIV distributions are categorized by vertebral region (cervical, upper thoracic, lower thoracic, lumbar, sacral/iliac) according to study-specific reporting. Surgical approach is reported as posterior, combined (anterior–posterior), or anterior where specified. “NR” denotes not reported

*3-CO* three-column osteotomy, *LIV* Lower instrumented vertebra, *MC* Mechanical complication, *PJF* Proximal junctional failure, *PJK* Proximal junctional kyphosis, *SD* Standard deviation, *UIV* Upper instrumented vertebra

Across studies, the proportion developing PJK/PJF ranged from 17.8% to 62.5%. Where reported, cohorts were predominantly older adults (mean age 65.4 years) and female (80.3%), a with a mean body mass index (BMI) of 25.9. The mean number of fused levels was 8.9. Follow-up ranged from 7.2 to 33.0 months. Geographically, studies originated from the United States (31.3%), China (31.3%), South Korea (18.8%), Japan (12.5%), and Spain (6.3%).

Definitions of PJK were reported in 14 studies (87.5%) and PJF in 9 studies (56.3%). PJK criteria were heterogeneous, most commonly defined as postoperative PJA ≥ 10° with ≥ 10° increase from baseline (7/14 studies), though thresholds ranged from > 10° to > 25°, with some incorporating SRS-Schwab modifier deterioration. PJF definitions generally reflected structural or mechanical failure, often coupled with revision surgery, with angular thresholds ranging from ≥ 10° to ≥ 20°.

Across studies reporting data partitions, 2869 patients were used for training or cross-validation, 764 for internal testing, and only 37 for external validation.

### Model characteristics, inputs, and validation approaches

Model- and workflow-level characteristics are summarized in Table 4.

**Table 4:** Machine learning model development, validation strategies, and feature domains across included studies

Author (year)	Algorithm(s)	Cross-validation strategy	Train:test:external validation split	Training sample size (n)		Training split (PJK/F:non-PJK/F:total)	Testing sample size (n)	Testing split (PJK/F:non-PJK/F:total)	External validation sample size (n)	External validation split (PJK/F:non-PJK/F:total)	Imaging modality	SHAP analysis	Calibration	Feature domains (underlined key predictors)
Scheer (2016)	DT	None	70:30:0	357		NR	153	NR	NR	NR	Radiographs (full-length, standing lateral)	No	NR	Demographics (age, sex, BMI) Surgical (UIV/LIV level, fused levels, implant type, fusion levels, 3CO, revision status) Radiographic (PT, PI-LL, LL, TK, SVA, coronal Cobb, SRS-Schwab)
Yagi (2018)	DT	None	70:30:0	112		22:90:112	33	NR	NR	NR	Radiographs (whole spine) DEXA	No	NR	Demographics (age, sex, BMI) Surgical (UIV/LIV level, fused levels, revision status, osteotomy) Bone quality (DEXA T-score) Radiographic (C7 SVA, PT, PI-LL, TPA, TK, SS, LL, SRS-Schwab)
Johnson (2023)	SVM CNN	fivefold	70:10:20	172		NR	19	NR	6	NR	Radiographs MRI	No	NR	Demographics (age, sex, BMI, comorbidities) Surgical (UIV/LIV level, fused levels, pelvic fixation, previous surgery) Radiographic (coronal: C7 plumbline, cervical major & minor curve apex deviation & Cobb angle, T1 tilt, thoracic curve apex deviation; sagittal: C2 slope, cervical lordosis, cervicothoracic-pelvic angle, L1-L4 angle, L1-S1 angle, L1 pelvic angle, L4-S1 angle, LL, PI, PI-LL, PT, SS, C2-C7 SVA, T1 spinopelvic inclination)
Lee (2023)	RF SVM LDA CART KNN	fivefold	70:30:0	154		35:119:154	47	14:33:47	NR	NR	Radiographs	No	NR	Demographics (age, BMI) Radiographic with SRS modifiers (curve pattern, PI, PI-LL, global balance, deformity type, PJA)
Ryu (2023)	LR DT RF GB	tenfold	70:30:0	147		41:106:147	63	17:46:63	NR	NR	Radiographs (Electronic Optical Scan)	Yes	NR	Demographics (age, sex, BMI) Surgical (index level, previous surgery, PJF) Bone quality (DEXA T-score) Radiographic (SVA, C7 SVA, PT, TK, T1 slope)
Tretiakov (2023)	LR	NR	70:30:0	545		NR	234	NR	NR	NR	Radiographs (SpineView)	No	NR	Demographics (age) HR-QoL indices (SF-36, ODI, SF-22, ASD frailty index) Surgical (PJK prophylaxis use, fused levels) Radiographic preoperative & 6-weeks with SAA modifier (TPA, PT, PI-LL)
Hiyama (2025)	LR SVM DT NB RF	fivefold tenfold	80:20:0	74		17:57:74	18	4:14:18	0	NR	Radiographs (full-length, AP & lateral)	No	NR	Demographics (age, sex, BMI) Surgical (LIV at sacrum, fusion levels) Radiographic (PI-LL, PT, TK, SS, LL, SVA)
Meng (2025)	LR LASSO NB SVM RF KNN MLP LightGBM	tenfold	NR	105		NR	NR	NR	37	NR	Radiographs (standing) CT MRI DEXA	No	NR	Demographics (age, sex, BMI, comorbidities, ASA grade, smoking) Surgical (fusion length) Bone quality (DEXA T-score, MRI-VBQ) Radiographic (PI, PT, LL, SS, SVA, TPA, Cobb angle)
Sundrani (2025)	SVM	fivefold	75:25:0	173		NR	58	NR	NR	NR	Radiographs MRI DEXA	Yes	NR	Demographics (age, sex, BMI, height, comorbidities) Bone quality (DEXA T-score) Radiographic (SVA, L1 angle, PT, CVA, L4-S1 angle) Sarcopenia (muscle-to-bone ratio, fatty infiltration)
Wang (2025)	LR DT KNN RF SVM GBM XGB NNET	fivefold	70:30:0	150		55:95:150	63	23:40:63	NR	NR	Radiographs CT MRI	Yes	NR	Demographics (age, sex, BMI, frailty) HR-QoL indices (symptom duration, WOMAC, SRS-22r) Bone quality (DEXA T-score) Surgical (UIV level, fused levels, operating time, estimated blood loss, 3-CO, bone cement) Radiographic (TK, LL, SS, PT, PI, SVA, PI-LL, pelvic compensation) Sarcopenia (fatty infiltration, rfCSA, rtCSA)
Li (2025)	GB DT RF LR GNB	tenfold	70:30:0	149		NR	64	26:38:64	NR	NR	Radiographs MRI	Yes	Brier score: RF (0.198), GNB (0.259), LR (0.244), GB (0.254), DT (0.286)	Demographics (age, BMI, comorbidities) Bone quality (MRI-VBQ) Surgical (UIV level, fused levels, osteotomy, pelvic fixation, TLIF) Sarcopenia (fatty infiltration, CSA) Radiographic (TPA, SVA, PT, SS, PI, LL, TK, Cobb angle, LDI, spinopelvic alignment)
Pizones (2024)	LR	NR	NR	391		NR	NR	NR	NR	NR	Radiographs (sagittal)	No	NR	Demographics (age, sex, BMI, ASA) HR-QoL indices (ODI, SRS-22, SF-36 PCS, SF-36 MCS) Surgical (approach, operative time, estimated blood loss, UIV level, osteotomy) Radiographic (PI, LL, TK, RSA, GT, RLL, DFI, RPV, SS, TSPA, T4PA, L1PA, LDI)
Li (2023)	MLR	None	NR	113		53:60:113	NR	NR	NR	NR	Radiographs (full-length, standing)	No	NR	Demographics (age, sex, BMI) Surgical (TK, PT, PI-LL, TK mismatch, LL mismatch)
Kim (2020)	LR	None	NR		70	32:38:70	NR	NR	NR	NR	Radiographs (SpineView)	No	NR	Demographics (age, sex) Radiographic (TPA, C2-T3 SVA, SVA)
Peng (2020)	MLP	LOOCV	NR		12	3:9:12	12	3:9:12	NR	NR	Radiographs CT	No	NR	Demographics (age, sex) Radiographic (PJA)
Hills (2022)	LR	Bootstrap resampling	NR		145	47:98:145	NR	NR	NR	NR	Radiographs CT	No	Calibration slope: LR (1), bootstrap validation (0.14), overfitting adjustment (0.86) Brier score: LR (0.096), bootstrap validation (−0.003), overfitting adjustment (0.10)	HR-QoL (CCI) Bone quality (CT-BMD) Radiographic (PI, TPA, L1-L4 lordosis, L4-S1 lordosis)

Summary of machine learning (ML) model development characteristics for studies predicting proximal junctional kyphosis (PJK), proximal junctional failure (PJF), or composite mechanical complications following adult spinal deformity surgery. For each study, algorithm(s), cross-validation strategy, dataset partitioning (train:test:external validation split), sample sizes with outcome breakdown, imaging modality, use of SHapley Additive exPlanations (SHAP) analysis, calibration assessment, and feature domains are reported. Feature domains are categorized as demographic, surgical, bone quality, radiographic, sarcopenia, and health-related quality-of-life (HR-QoL) variables. Where applicable, key predictors identified in the best-performing model are underlined within the table. “NR” denotes not reported

*3-CO* three-column osteotomy, *AP* Anteroposterior, *ASA* American Society of Anesthesiologists classification, *ASD* Adult spinal deformity, *BMI* Body mass index, *CART* classification and regression tree, *CCI* Charlson Comorbidity Index, *CSA* Cross-sectional area, *CT* Computed tomography, *CT-BMD* Computed tomography–derived bone mineral density, *CV* Cross-validation, *CVA* Coronal vertical axis, *DEXA* Dual-energy X-ray absorptiometry, *DT* Decision tree, *GB* Gradient boosting, *GBM* Gradient boosting machine, *GNB* Gaussian naïve Bayes, *HR-QoL* Health-related quality of life, *KNN* k-nearest neighbour, *LASSO* Least absolute shrinkage and selection operator, *LDA* Linear discriminant analysis, *LL* Lumbar lordosis, *LOOCV* Leave-one-out cross-validation, *LR* Logistic regression, *MC* Mechanical complication, *MLP* Multilayer perceptron, *MLR* Multiple logistic regression, *MRI* Magnetic resonance imaging, *NNET* Neural network, *ODI* Oswestry Disability Index, *PI* Pelvic incidence, *PI–LL* Pelvic incidence–lumbar lordosis mismatch, *PJA* Proximal junctional angle, *PJF* Proximal junctional failure, *PJK* Proximal junctional kyphosis, *PT* Pelvic tilt, *RF* Random forest, *rFCSA* Relative functional cross-sectional area, *rTCSA* Relative total cross-sectional area, *RSA* Relative spinopelvic alignment, *SAAS* Sagittal age-adjusted score, *SHAP* SHapley Additive exPlanations, *SRS-22* Scoliosis Research Society-22 questionnaire, *SVA* Sagittal vertical axis, *TK* Thoracic kyphosis, *TLIF* Transforaminal lumbar interbody fusion, *TPA* T1 pelvic angle, *UIV* Upper instrumented vertebra, *VBQ* Vertebral bone quality score, *WOMAC* Western Ontario and McMaster Universities Osteoarthritis Index, *XGB* Extreme gradient boosting

Common algorithms logistic regression (30.0%), random forest (20.0%), support vector machine (20.0%), and decision tree-based approaches (20.0%). Cross-validation was performed in 10 studies [12, 13, 15, 22, 24, 28, 32, 35, 45, 48], most frequently using a 70:30 train-test split (7 studies). External validation was rare (1 study).

Radiographic parameters were reported in all 16 studies (100.0%) [12, 13, 15, 17, 22, 24, 25, 28, 32, 33, 35, 38, 45, 46, 48, 50]. Bone quality metrics (such as dual-energy X-ray absorptiometry (DEXA)-based bone mineral density (DEXA-BMD), computed tomography (CT)-based Hounsfield units (CT-HUs), and magnetic resonance imaging (MRI)-based vertebral bone quality (MRI-VBQ)) were incorporated in 7 studies (43.8%) [12, 24, 28, 35, 45, 48, 50], and sarcopenia or muscle-quality metrics in 3 studies (18.8%) [24, 45, 48]. Shapley Additive exPlanations (SHAP)-based interpretability was reported in 4 studies (25.0%) [24, 35, 45, 48]. Calibration was inconsistently assessed.

Prediction targets included PJK incidence (10 studies [12, 15, 22, 25, 28, 32, 33, 38, 46, 48]), PJF incidence (4 studies [13, 24, 45, 50]), composite mechanical complications (2 studies [24, 45]), PJK recurrence (1 study [17]), PJK severity scoring (1 study [12]), and unplanned reoperation (1 study [35]).

### Diagnostic test accuracy meta-analysis

Twenty-two models [13, 22, 24, 25, 32, 35, 48] provided extractable test-set confusion matrixes and were included in diagnostic pooling.

**Fig. 2:**
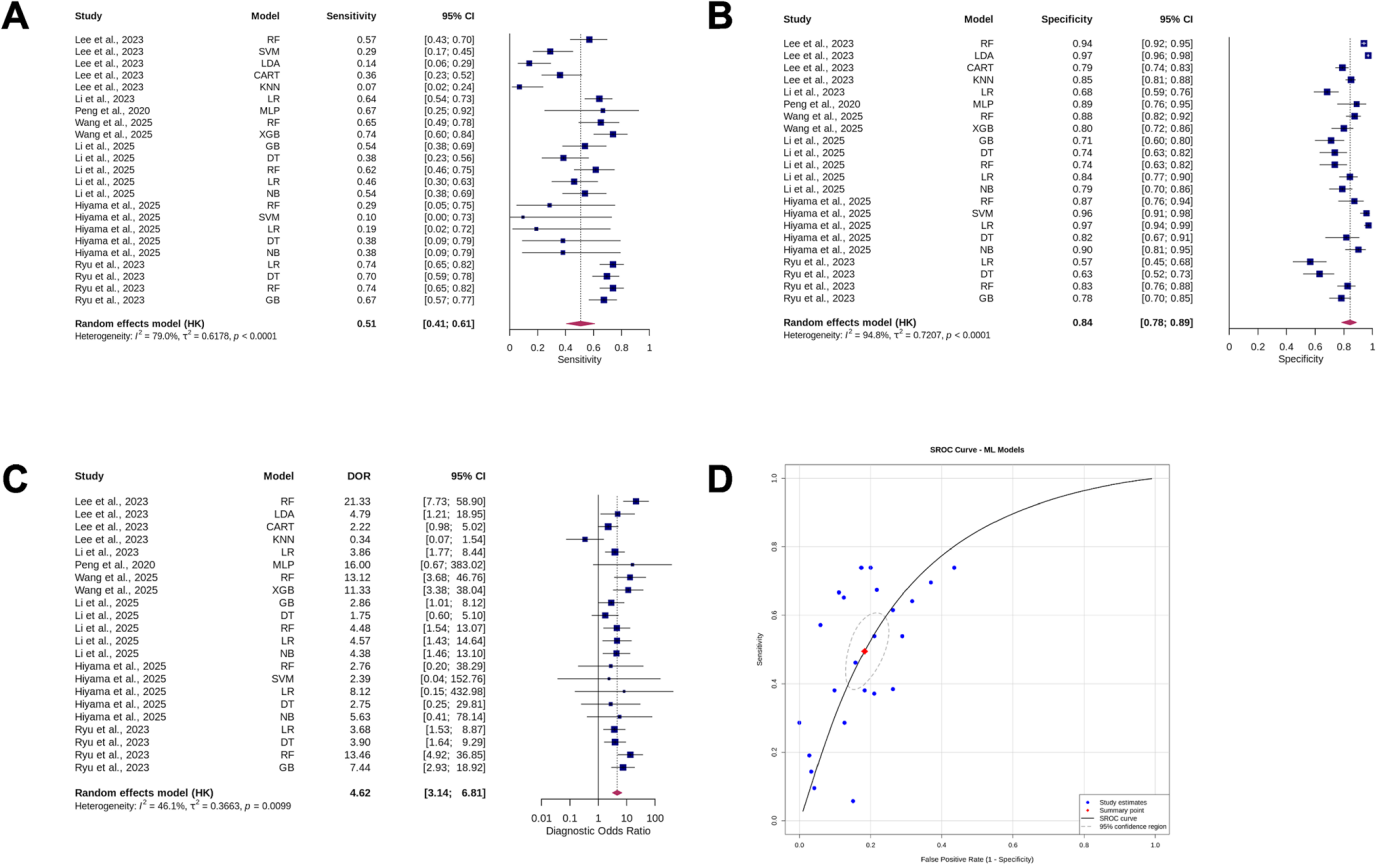
Diagnostic performance of all included machine learning models for prediction of proximal junctional pathology. Forest plots generated using random-effects models demonstrating pooled (**A**) sensitivity and (**B**) specificity, (**C**) diagnostic odds ratio (dOR), and (**D**) summary receiver operating characteristic (SROC) curves for all models with available test-set confusion matrices (k = 22). Squares represent individual model estimates weighted by inverse variance, with horizontal lines denoting 95% confidence intervals. Diamonds indicate pooled estimates. The SROC curve depicts overall discrimination derived from the bivariate framework, with corresponding confidence region

Pooled sensitivity was 0.51 (95% CI: 0.41–0.61; I^2^ = 79.0%) and pooled specificity was 0.84 (95% CI: 0.78–0.89; I^2^ = 94.8%). The pooled dOR of 4.62 (95% CI: 3.14–6.81; I^2^ = 46.1%). SROC analysis yielded a pooled AUC of 0.67 (95% CI: 0.62–0.72) (Fig. 2). Overall, ML models demonstrated modest discrimination with higher specificity than sensitivity.

**Fig. 3:**
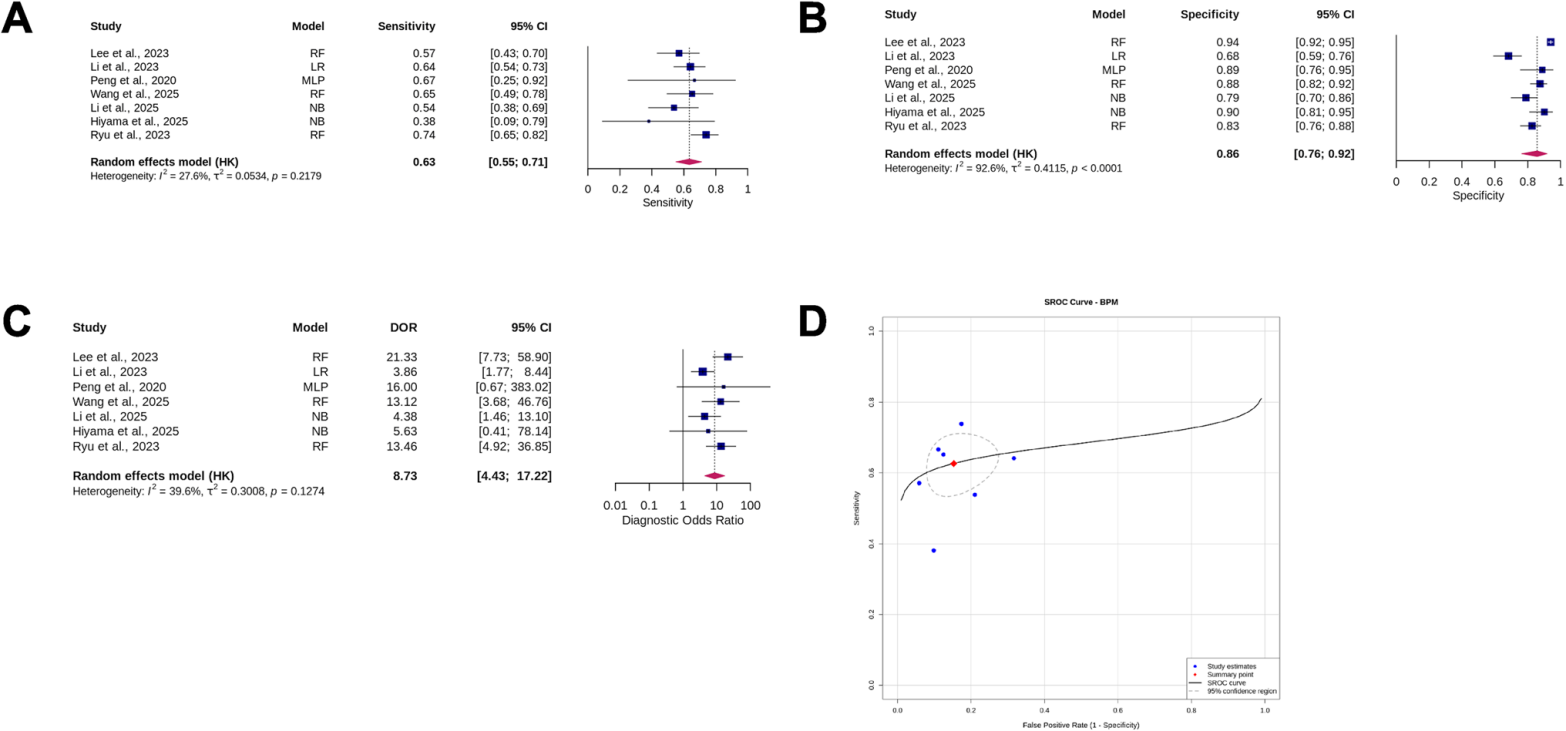
Diagnostic performance of the highest-performing machine learning model per study for prediction of proximal junctional pathology. Forest plots generated using random-effects models demonstrating pooled (**A**) sensitivity and (**B**) specificity, **C** diagnostic odds ratio (dOR), and (**D**) summary receiver operating characteristic (SROC) curves using one highest performing model per study to reduce within-study dependence. Squares represent individual model estimates weighted by inverse variance, with horizontal lines denoting 95% confidence intervals. Diamonds indicate pooled estimates. The SROC curve summarizes discrimination across studies within the bivariate diagnostic accuracy framework

To reduce within-study dependence, a prespecified analysis pooling one best-performing model per study showed pooled sensitivity improved to 0.63 (95% CI: 0.55–0.71; I^2^ = 27.6%), and specificity remained high at 0.86 (95% CI: 0.76–0.92; I^2^ = 92.6%). The pooled dOR increased to 8.73 (95% CI: 4.43–17.22; I^2^ = 39.6%), with overall accuracy of 0.73 (95% CI 0.69–0.76, I^2^ = 0.5%) and pooled AUC of 0.77 (95% CI: 0.71–0.83; I^2^ = 38.0%) (Fig. 3). These results represent the upper bound of performance within the current evidence base.

### Clinical utility

Using pooled likelihood ratios (PLR 2.71; NLR 0.62), Fagan nomogram analysis demonstrated that, assuming a 20.0% pre-test probability, a positive ML classification increased post-test probability to 40.5%, whereas a negative classification reduced it to 13.4% (Supplementary Fig. 1). Thus, ML predictions shifted risk probability but did not provide rule-in or rule-out performance sufficient for categorical decision-making.

### Predictors of proximal junctional pathology

Across studies reporting feature importance, predictors consistently clustered into four domains: (i) patient factors, with age (most consistent), BMI, and frailty indices; (ii) sagittal alignment parameters, including sagittal vertical axis (SVA), pelvic tilt (PT), and pelvic incidence-lumbar lordosis (PI–LL) mismatch; (iii) construct & surgical factors, including UIV and lower instrumented vertebra (LIV) level, fusion length, osteotomy use, prophylaxis, and implant strategy; (iv) tissue quality metrics, include DEXA-BMD, CT-HU, MRI-VBQ, and paraspinal muscle fatty infiltration. Several models incorporated postoperative or early postoperative alignment variables (such as postoperative PJA, 6-week modifiers), improving apparent discrimination but limiting pure preoperative risk stratification.

High-performing examples included Yagi et al. (2018) [50] (decision-tree AUC 1.00), Tretiakov et al. (2023) [46] (linear regression AUC 0.92), Scheer et al. (2016) [38] (decision-tree AUC 0.89), and Lee et al. (2023) [22] (random forest AUC 0.76). These models consistently emphasized age, sagittal alignment, fusion extent, UIV characteristics, and bone quality proxies.

### Publication bias, risk of bias, and certainty of evidence

Deeks' funnel plot asymmetry test showed no evidence of significant small-study effects (*P* = 0.88) (Supplementary Fig. 2). Overall risk of bias (PROBAST) was rated as unclear to low in most studies, primarily due to limited reporting of calibration and external validation (Supplementary Fig. 3). Certainty of evidence (GRADE) for pooled diagnostic accuracy outcomes was rated as moderate, with downgrading due to heterogeneity (Supplementary Table 2).

## Discussion

### Machine learning and junctional pathology: promise versus performance

Proximal junctional pathology remains one of the most consequential and least predictable complications following ASD surgery. Despite decades of biomechanical study and risk-factor analysis, surgeons still face substantial uncertainty when counselling patients about the likelihood of PJK or PJF [9, 30]. ML has emerged within this context as a proposed solution – promising to integrate complex multivariable interactions beyond the capacity of traditional regression modelling.

In this systematic review and diagnostic meta-analysis, ML-based models demonstrated statistically meaningful but clinically moderate discrimination. Pooled performance across all models yielded an AUC of 0.67, improving to 0.77 when restricted to the highest-performing model per study. Specificity was consistently higher than sensitivity, indicating greater ability to identify high-risk patients than to exclude future events. These findings suggest that ML is capturing real biological and mechanical structure within the data, but they also underscore a central tension: current models refine risk estimates without yet resolving the fundamental unpredictability of junctional pathology.

Rather than representing a paradigm shift, ML presently functions an incremental extension of established biomechanical reasoning. The key question is therefore not whether ML can detect signal – it can – but whether that signal is sufficiently stable, transportable, and decision-relevant to alter surgical strategy.

### Biomechanical foundations of proximal junctional pathology

Interpretation of these findings requires situating junctional pathology within its established biomechanical framework. PJK was originally defined radiographically as ≥ 10° increase in PJA following long posterior constructs and has since been understood as a mechanical consequence of abrupt transition from rigid instrumentation to mobile adjacent segments. Subsequent work has demonstrated that long-segment fusion alters load transfer, concentrates stress at the UIV and supra-adjacent segment, and increases susceptibility to structural compromise when substantial sagittal correction is imposed [51]. Whether this mechanical stress manifests as isolated angular progression (PJK) or structural failure with vertebral fracture, implant pull-out, or need for revision (PJF) depends on the interaction between corrective demand and biological reserve [7]. Junctional failure is therefore not a discrete event but the culmination of stress redistribution exceeding local structural tolerance [14, 51].

The predictors most consistently prioritized across included ML studies reflect this interactional paradigm. Sagittal alignment variables (including SVA, PT, and PI-LL mismatch) encode deformity magnitude and corrective force [20, 40]. Construct characteristics (including fusion length, osteotomy grade, UIV selection, and pelvic fixation) modulate stiffness gradients and moment transfer [10]. Measures of bone quality (including DEXA-BMD, CT-HU, MRI-VBQ) approximate vertebral body strength and resistance to compressive and tensile fracture [3]. Age, BMI, and frailty markers contextualize systemic physiological resilience. Mechanistically, these features converge on a permissive state in which mechanical demand outstrips structural capacity. In this sense, ML models appear to integrate known determinants into higher-dimensional representations of biomechanical-biological interaction rather than identifying entirely novel risk domains.

### Endpoint heterogeneity and structural limits to discrimination

The moderate pooled discrimination observed in this analysis likely reflects limitations of the underlying evidence base. Definitions of PJK varied substantially across studies, ranging from ≥ 10° to ≥ 25° PJA threshold, sometimes incorporating interval change criteria or SRS-Schwab modifier deterioration. PJF definitions were even more heterogeneous, combining radiographic parameters with clinical failure or revision surgery. Predicting mild radiographic progression at one year is fundamentally different from predicting structural collapse requiring reoperation. Pooling across these distinct biological and clinical entities introduces label heterogeneity that inevitably attenuates discrimination.

Threshold dependence further complicates interpretation. Most ML models generate continuous risk probabilities, yet diagnostic synthesis requires dichotomization. In several studies, classification thresholds were selected in-sample to optimize performance and were inconsistently reported. Minor variations in cut-off selection can materially alter sensitivity and specificity. Without prespecified thresholds and transparent calibration reporting, inter-study differences may reflect analytic choices as much as biological signal.

### Transportability and validation

External validation was limited across the included literature. Notably, several models reported near-perfect discrimination (AUC ≈ 1.0), which is biologically implausible in this context and strongly suggests overfitting. These estimates likely reflect optimisation within small, internally validated datasets rather than true predictive signal. In the absence of external validation, such performance should be interpreted as optimistic and unlikely to be reproducible across independent cohorts. Most studies relied on internal train-test splits or cross-validation, with minimal independent cohort testing. Given recognized inter-center variation in deformity severity, correction targets, osteotomy utilization, prophylactic strategies, and revision thresholds, dataset shift is expected [16]. Models trained within a single institution ecosystem may encode local practice patterns alongside genuine biological risk. Without robust multicenter validation, internal performance metrics risk overestimating real-world transportability.

### Preoperative prediction versus early trajectory modelling

A further conceptual tension concerns the definition of prediction itself. Some models incorporated early postoperative variables, such as early PJA change or alignment modifier deterioration. These features lie on the causal pathway toward junctional pathology and function more as trajectory markers than true preoperative predictors. While their inclusion improves apparent discrimination, it alters the clinical question being addressed. Preoperative models inform planning and counselling; postoperative models detect evolving biomechanical drift. Conflating these use cases may inflate performance estimates while obscuring translational relevance.

### Operating characteristics and clinical role

The pooled operating profile – relatively high specificity and limited sensitivity – suggests that current ML models are more conservative in identifying high-risk patients than they are effective in excluding future pathology. Clinically, this pattern supports a role in risk stratification rather than screening. A high-risk classification may justify intensified surveillance or consideration of junction-protective strategies, whereas a low-risk classification does not reliably obliviate such measures. ML presently refines probabilistic counselling but does not independently determine construct design, prophylaxis, or operative indication.

Importantly, performance did not appear consistently superior for complex neural network architectures relative to tree-based methods or penalized regression approaches. This aligns with broader observations in clinical prediction modelling, where endpoint clarity, feature definition, and data quality often outweigh classifier complexity [2, 4]. In ASD surgery, the dominant predictive signal appears embedded within established biomechanical variables rather than latent nonlinear structures requiring deep representation learning.

### Translational implications and future direction

ML-based models currently offer incremental rather than transformative clinical value. They may enhance shared decision-making by refining risk estimates and potentially guide surveillance intensity. However, no available model demonstrates rule-in or rule-out performance sufficient to independently dictate operative strategy or prophylactic omission.

Advancement toward clinically deployable decision support will require harmonized endpoint definitions distinguishing radiographic PJK from clinically consequential PJF, prespecified thresholds, consistent calibration reporting, and rigorous external validation across heterogeneous practice environments [5]. Decision-curve analysis and net-benefit modelling will be essential to determine whether statistical improvement translates into meaningful clinical advantage [2, 47]. Notably, none of the included studies employed segmentation-based radiomic pipelines, with most models relying instead on structured clinical and radiographic features. While radiomics may enable extraction of higher-dimensional imaging signatures, its application in this domain remains limited, and its incremental value over established biomechanical variables is yet to be demonstrated.

### Limitations

This meta-analysis is limited by the retrospective design of all included studies, heterogeneous endpoint definitions, and variable reporting of confusion matrices and calibration metrics. Pooling multiple models per cohort introduces potential non-independence, although secondary highest-performing-model analysis mitigated this effect. As with all diagnostic syntheses of prediction models, threshold harmonization imposes unavoidable approximation.

More broadly, synthesis of prediction model performance presents inherent methodological challenges. Included studies varied substantial in feature selection, modelling strategy, outcome definition, and validation approach. While diagnostic test accuracy methods allow aggregation of classification performance, such pooling necessarily abstracts models from their underlying structure and should be interpreted as an estimate of overall performance bounds rather than direct model comparability. Established methodological frameworks for prediction model synthesis [6, 29] emphasize the importance of calibration, transportability, and consistent reporting, domains that were variably addressed across the included literature.

The search strategy was designed to balance sensitivity and specificity for ML-based prediction of proximal junctional pathology. However, the use of targeted search terms may have limited retrieval of some potentially relevant studies, particularly given variability in terminology used to describe ML approaches and proximal junctional outcomes across the literature.

## Conclusion

ML-based prediction of proximal junctional pathology after ASD surgery demonstrates biologically coherent but statistically moderate discrimination. The determinants identified across studies align with a mechanistic paradigm in which junctional failure emerges from interaction between corrective biomechanics and biological reserve. Current models function as risk-modifying adjuncts rather than definitive classifiers. Progress toward clinically actionable decision support will depend less on algorithmic escalation and more on standardization, calibration, and robust external validation. Until then, ML should be regarded as an extension of biomechanical reasoning rather than its replacement.

Summary of included retrospective cohort (RC) studies evaluating machine learning (ML) models for prediction of proximal junctional kyphosis (PJK), proximal junctional failure (PJF), or composite mechanical complications (MC) following adult spinal deformity (ASD) surgery. For each study, country of origin, follow-up duration, cohort size (PJK/PJF, non-PJK/PJF, and total), event rate, radiographic definitions of ASD and proximal junctional pathology, proximal junctional angle (PJA) measurement method, inclusion criteria, and primary outcome predicted are reported. Where applicable, PJA was calculated as the sagittal Cobb angle between the inferior endplate of the upper instrumented vertebra (UIV) and the superior endplate of UIV + 2. “NR” denotes not reported.

Baseline patient demographics, bone quality metrics, and preoperative radiographic parameters across included studies, stratified by proximal junctional kyphosis (PJK), proximal junctional failure (PJF), or composite mechanical complication status where reported. Continuous variables are presented as mean ± standard deviation (SD) or median (range/interquartile range) as originally reported. Radiographic alignment parameters include sagittal vertical axis (SVA), pelvic tilt (PT), pelvic incidence–lumbar lordosis mismatch (PI–LL), pelvic incidence (PI), and SRS-Schwab coronal curve type and global balance modifiers. Where applicable, SRS-Schwab modifiers are reported as 0 (no deformity), + (moderate deformity), or + + (severe deformity). “NR” denotes not reported.

Summary of operative characteristics for included cohorts, including number of primary and revision surgeries, fusion length, upper instrumented vertebra (UIV) and lower instrumented vertebra (LIV) distribution, three-column osteotomy (3-CO) rates, implant type, and surgical approach. Where reported, values are stratified by proximal junctional kyphosis (PJK), proximal junctional failure (PJF), or composite mechanical complication status. Fusion length is presented as mean ± standard deviation (SD) or median (range) as originally reported. UIV and LIV distributions are categorized by vertebral region (cervical, upper thoracic, lower thoracic, lumbar, sacral/iliac) according to study-specific reporting. Surgical approach is reported as posterior, combined (anterior–posterior), or anterior where specified. “NR” denotes not reported.

Summary of machine learning (ML) model development characteristics for studies predicting proximal junctional kyphosis (PJK), proximal junctional failure (PJF), or composite mechanical complications following adult spinal deformity surgery. For each study, algorithm(s), cross-validation strategy, dataset partitioning (train:test:external validation split), sample sizes with outcome breakdown, imaging modality, use of SHapley Additive exPlanations (SHAP) analysis, calibration assessment, and feature domains are reported. Feature domains are categorized as demographic, surgical, bone quality, radiographic, sarcopenia, and health-related quality-of-life (HR-QoL) variables. Where applicable, key predictors identified in the best-performing model are underlined within the table. “NR” denotes not reported.

## Supplementary Information

Below is the link to the electronic supplementary material.

ESM 1 Supplementary Material 1 (DOCX 823 KB)

## Data Availability

No datasets were generated or analysed during the current study.

## Abbreviations

ASD: Adult Spinal Deformity
AUC: Area Under the Curve
AI: Artificial Intelligence
CT: Computed Tomography
CT-HU: Computed Tomography-Based Hounsfield Unit
CI: Confidence Interval
dOR: Diagnostic Odds Ratio
DEXA: Dual-Energy X-ray Absorptiometry
DEXA-BMD: Dual-Energy X-ray Absorptiometry-Based Bone Mineral Density
LIV: Lower Instrumented Vertebra
ML: Machine Learning
MRI: Magnetic Resonance Imaging
MRI-VBQ: Magnetic Resonance Imaging-Based Vertebral Bone Quality
NLR: Negative Likelihood Ratio
PI–LL: Pelvic Incidence-Lumbar Lordosis
PT: Pelvic Tilt
PLR: Positive Likelihood Ratio
PJA: Proximal Junctional Angle
PJF: Proximal Junctional Failure
PJK: Proximal Junctional Kyphosis
SVA: Sagittal Vertical Axis
SROC: Summary Receiver Operating Curve
UIV: Upper Instrumented Vertebra
