# Supplementary material for: Machine learning-based prediction of proximal junctional pathology after adult spinal deformity surgery: a systematic review and diagnostic test accuracy meta-analysis
Source: Acta Neurochir (Wien). 2026 May 7;168(1):148. doi: 10.1007/s00701-026-06876-6 (PMC13319844; doi:10.1007/s00701-026-06876-6)
Supplement: Supplementary file 1 — Supplementary Material 1 (DOCX 823 KB) [file 701_2026_6876_MOESM1_ESM.docx]

| **Database** | **Search Strategy** |
| --- | --- |
| PubMed  (n=30) | ("Kyphosis"[Mesh] OR "proximal junctional kyphosis"[tiab] OR "proximal junctional failure"[tiab]  OR "proximal junctional"[tiab] OR "PJK"[tiab] OR "PJF"[tiab] OR "junctional kyphosis"[tiab]  OR "junctional failure"[tiab])  AND  ("Machine Learning"[Mesh] OR "Artificial Intelligence"[Mesh] OR "Deep Learning"[Mesh]  OR "Neural Networks, Computer"[Mesh] OR "machine learning"[tiab] OR "deep learning"[tiab]  OR "artificial intelligence"[tiab] OR "neural network*"[tiab] OR "support vector machine*"[tiab]  OR "random forest"[tiab] OR "decision tree"[tiab] OR "gradient boosting"[tiab]  OR "transformers"[tiab] OR "large language model"[tiab])  AND  ("Prognosis"[Mesh] OR "Risk Assessment"[Mesh] OR "predict*"[tiab] OR "risk prediction"[tiab]  OR "risk stratification"[tiab] OR "prediction model*"[tiab] OR "predictive model*"[tiab]  OR "prognosis"[tiab] OR "classification"[tiab]) |
| Embase  (n=45) | exp kyphosis/ OR (proximal junctional kyphosis OR proximal junctional failure OR proximal junctional  OR PJK OR PJF OR junctional kyphosis OR junctional failure).ti,ab,kw.  exp machine learning/ OR exp artificial intelligence/ OR exp deep learning/  OR exp artificial neural network/ OR exp support vector machine/ OR exp decision tree/  OR (machine learning OR deep learning OR artificial intelligence OR neural network*  OR support vector machine* OR random forest OR decision tree OR gradient boosting  OR transformers OR large language model).ti,ab,kw.  exp prognosis/ OR exp risk assessment/ OR exp prediction/  OR (predict* OR risk prediction OR risk stratification OR prediction model*  OR predictive model* OR prognosis OR classification).ti,ab,kw.  1 AND 2 AND 3 |
| CENTRAL  (n=2) | ID Search  #1 [mh "Kyphosis"]  #2 ("proximal junctional kyphosis" OR "proximal junctional failure" OR "proximal junctional" OR "PJK" OR "PJF" OR "junctional kyphosis" OR "junctional failure"):ti,ab,kw  #3 #1 OR #2  #4 [mh "Machine Learning"] OR [mh "Artificial Intelligence"] OR [mh "Deep Learning"] OR [mh "Neural Networks, Computer"]  #5 ("machine learning" OR "deep learning" OR "artificial intelligence" OR (neural NEXT network*) OR (support NEXT vector NEXT machine*) OR "random forest" OR "decision tree" OR "gradient boosting" OR "transformers" OR "large language model"):ti,ab,kw  #6 #4 OR #5  #7 [mh "Prognosis"] OR [mh "Risk Assessment"]  #8 (predict* OR "risk prediction" OR "risk stratification" OR (prediction NEXT model*) OR (predictive NEXT model*) OR "prognosis" OR "classification"):ti,ab,kw  #9 #7 OR #8  #10 #3 AND #6 AND #9 |

**Supplementary Table 1*:* Search Strategies**

Full electronic search strategies used to identify relevant studies for this review, reported for each database. n = number of articles returned.

| Algorithm | Factors that may decrease certainty of evidence | | | | | Performance | | Certainty of evidence |
| --- | --- | --- | --- | --- | --- | --- | --- | --- |
|  | Risk of bias | Indirectness | Inconsistency | Imprecision | Publication bias | Sensitivity | Specificity |  |
| ML | serious^1^ | not serious | not serious | not serious | none | 0.51 (0.41–0.61) | 0.84 (0.78–0.89) | ⨁⨁⨁◯ Moderate |
| BPM ML | serious^1^ | not serious | not serious | not serious | none | 0.63 (0.55–0.71) | 0.86 (0.76–0.92) | ⨁⨁⨁◯ Moderate |

^1^4 ‘unclear’ and 3 ‘low’ risk of bias on PROBAST

**Supplementary Table 2: Certainty of evidence (GRADE)**

GRADE assessment of certainty of evidence for the present review. ML, machine learning; BPM, best-performing model.


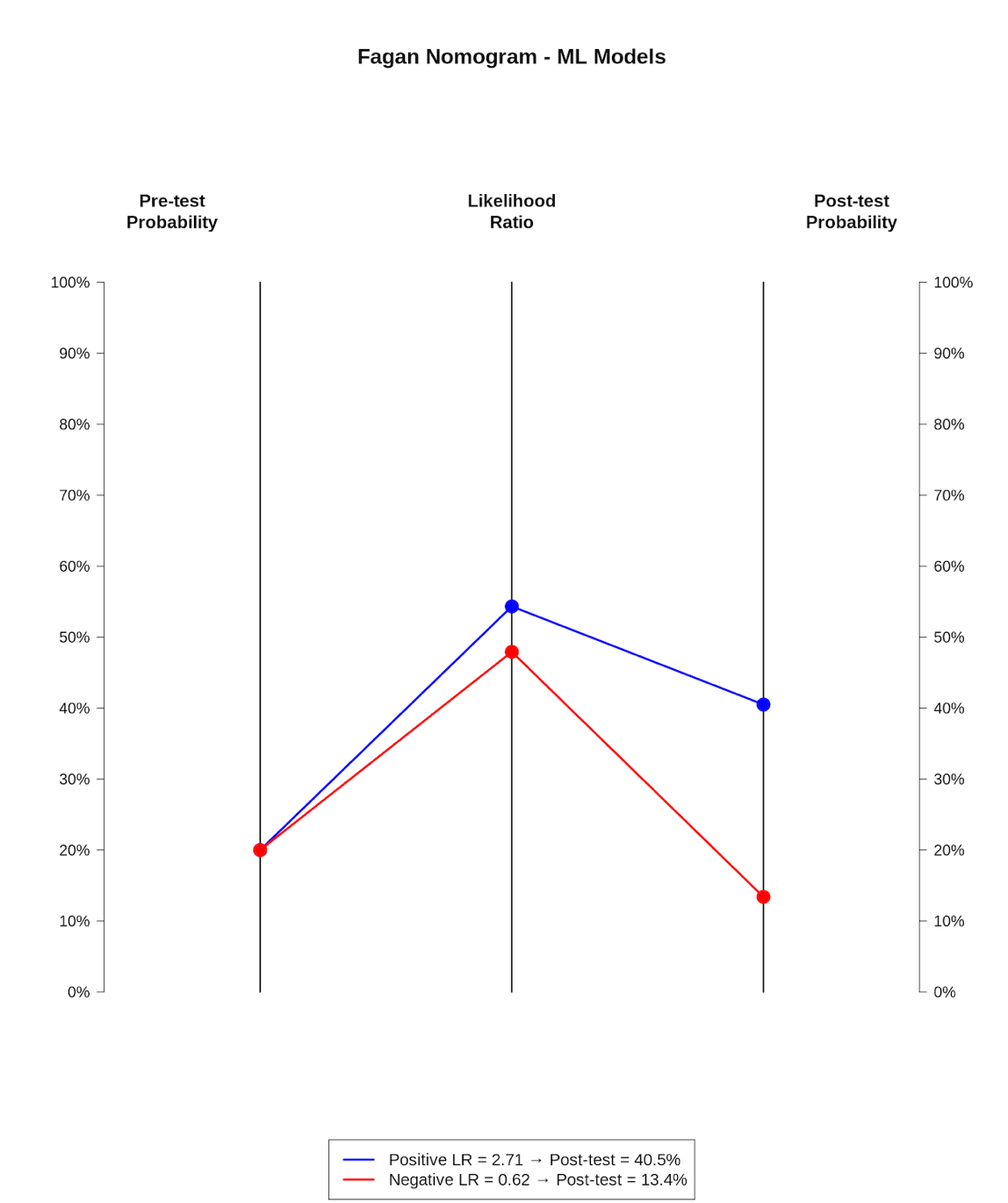


**Supplementary Figure 1: Fagan nomogram**

Fagan plots demonstrating the change from pre-test to post-test probability of proximal junctional pathology using pooled likelihood ratios for machine learning (ML) models. The blue line indicates the post-test probability following a positive ML classification, while the red line indicates the post-test probability following a negative ML classification.


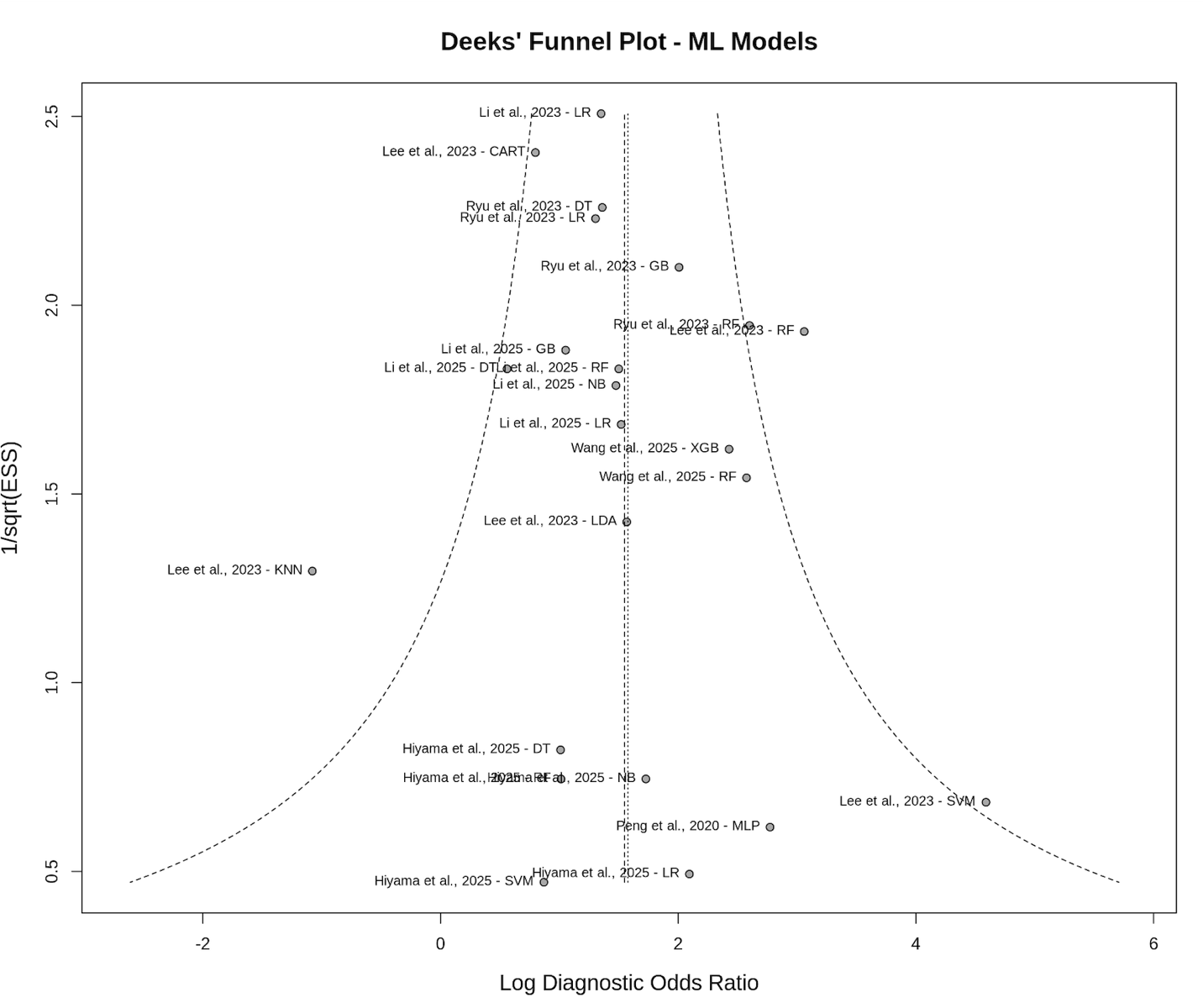


**Supplementary Figure 2: Publication bias assessment**

Deeks’ funnel plot for assessment of publication bias. Diagnostic odds ratio (log scale) is plotted against the inverse square root of the effective sample size. Circles represent individual studies, and the dashed line denotes the weighted linear regression line used in Deeks’ asymmetry test.


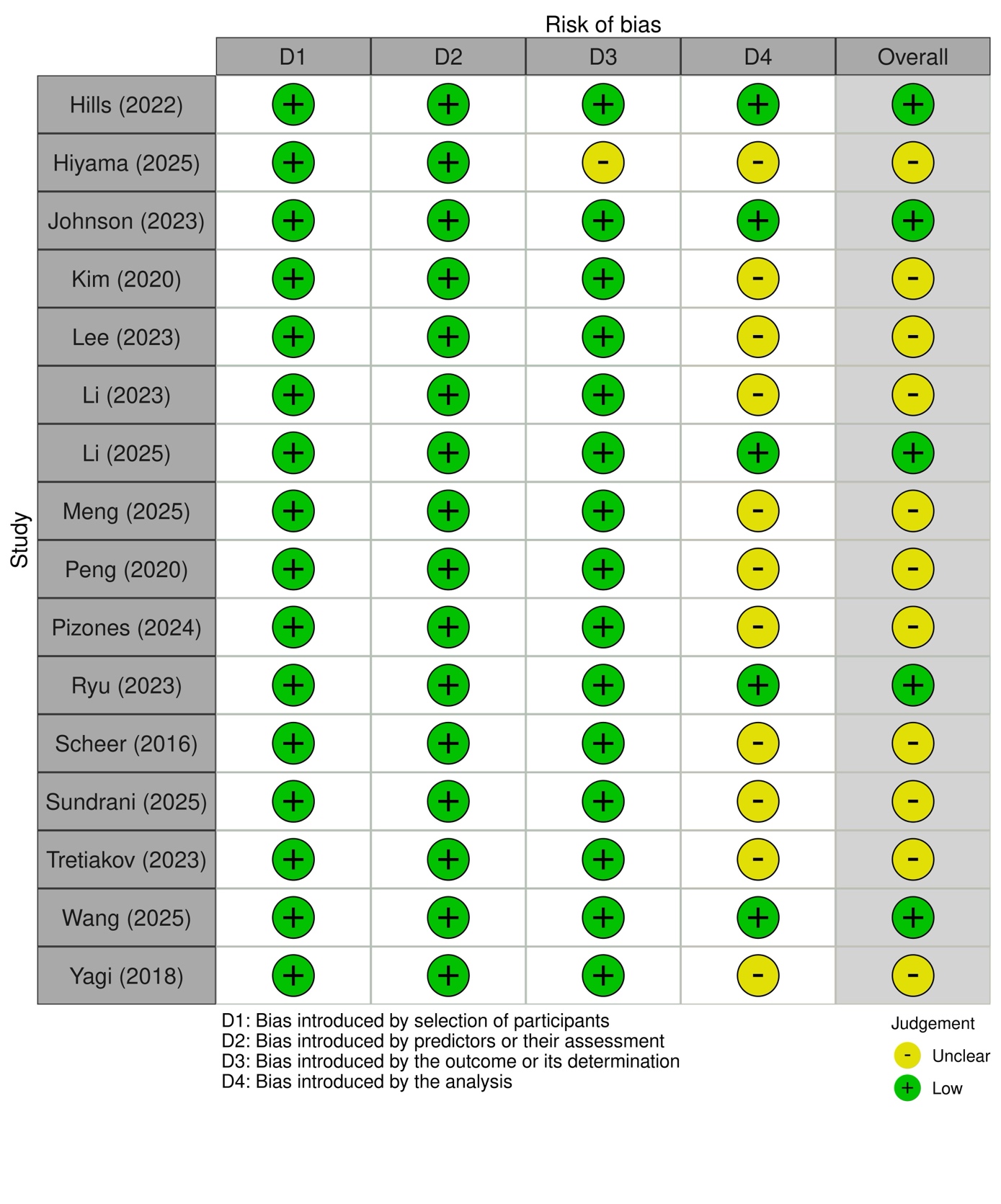


**Supplementary Figure 3: Risk of bias assessment**

Summary of risk of bias assessment for predictive models (PROBAST), reported by individual domain and overall risk of bias.
